# Thermoplastic Composites for Integrally Woven Pressure Actuated Cellular Structures: Design Approach and Material Investigation

**DOI:** 10.3390/polym13183128

**Published:** 2021-09-16

**Authors:** Michael Vorhof, Cornelia Sennewald, Philipp Schegner, Patrick Meyer, Christian Hühne, Chokri Cherif, Michael Sinapius

**Affiliations:** 1 Institute of Textile Machinery and High Performance Material Technology, Technische Universität Dresden, 01062 Dresden, Germany; cornelia.sennewald@tu-dresden.de (C.S.); philipp.schegner@tu-dresden.de (P.S.); chokri.cherif@tu-dresden.de (C.C.); 2 Institute of Mechanics and Adaptronics, Technische Universität Braunschweig, Langer Kamp 6, 38106 Braunschweig, Germany; pat.meyer@tu-braunschweig.de (P.M.); m.sinapius@tu-braunschweig.de (M.S.); 3 Institute of Composite Structures and Adaptive Systems, German Aerospace Center, Lilienthalplatz 7, 38108 Braunschweig, Germany; christian.huehne@dlr.de

**Keywords:** pressure-actuated cellular structure, shape morphing, compliant mechanism, anisotropic flexure hinges, textile-reinforced polymer composite, integrally woven structure

## Abstract

The use of pressure-actuated cellular structures (PACS) is an effective approach for the application of compliant mechanisms. Analogous to the model in nature, the Venus flytrap, they are made of discrete pressure-activated rows and can be deformed with high stiffness at a high deformation rate. In previous work, a new innovative approach in their integral textile-based manufacturing has been demonstrated based on the weaving technique. In this work, the theoretical and experimental work on the further development of PACS from simple single-row to double-row PACS with antagonistic deformation capability is presented. Supported by experimental investigations, the necessary adaptations in the design of the textile preform and the polymer composite design are presented and concretized. Based on the results of pre-simulations of the deformation capacity of the new PACS, their performance was evaluated, the results of which are presented.

## 1. Introduction

Compliant mechanisms offer immense advantages in their use. In contrast to conventional mechanisms, complex assemblies can be combined into a single part with the same functionality, and the mass and the number of necessary parts are significantly reduced. Components for coupling and guiding the individual parts are eliminated, as is the need for their maintenance and lubrication [1]. In general, the purpose of most mechanisms or mechanical systems is indirectly the transmission of energy or information between various interfaces. In addition to distribute the necessary material and energy flows, they make this possible directly by converting and transmitting forces and comparable mechanical quantities. In addition to the necessary energy sources and to an application-specific periphery, conventional mechanisms are made up of several links and coupling elements (joints). While the links are considered as idealized stiff structures, the joints have degrees of freedom (e.g., rotatory, translatory) so that kinematically determined mechanisms can be represented from their specific combination [2]. The advantage of this approach is that the kinematics and the stability (e.g., against mechanical and other loads) can be evaluated separately. This contrasts with the approach of using mechanisms that consist of only a single element and whose kinematic characteristics, e.g., their operational behavior, are represented by a locally differentiated compliance. The prerequisite for this is that the kinematics and the mechanical response (stresses, deformations) are considered together. The effort in the development of such compliant mechanisms is, therefore, depending on the application, significantly higher than for conventional mechanisms [3].

**Figure 1:**
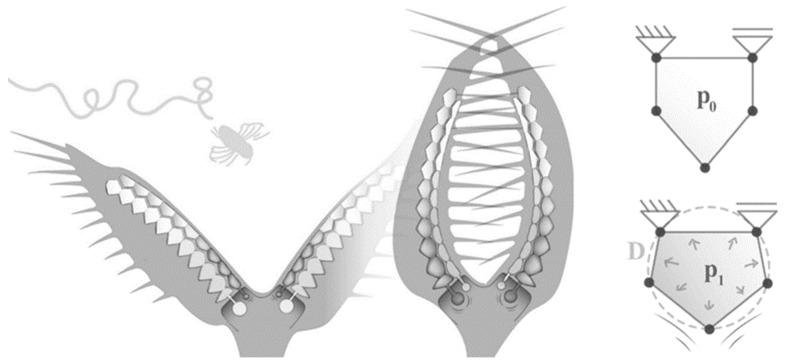
Schematic cross-sectional view of Venus flytrap and derived compliant mechanism of a single PACS Cell. Reprinted/adapted by permission from Springer Nature [11].

However, the advantages in the use of compliant mechanisms make them particularly interesting for aerospace applications. Shape-flexible structures and components made of fiber-reinforced composites enable mass savings but can also achieve significantly improved aerodynamic efficiency, for example, as a component of morphing wing technologies. Examples of this are the work on the ‘FlexFoil system’ (Kota et al. [4]), the ‘vertebrate structure’ (Elzey et al. [5]), or the ‘droop nose’ (Vasista et al. [6]). However, the problem with these concepts is the system-inherent contradiction between the required compliance and the necessary structural stiffness and strength. The stiffness is of particular importance for the creation of an accurately controlled aerodynamic shape. Cellular compliant structures with integrated pneumatic or hydraulic actuation have been identified as a promising solution, e.g., in [7,8,9]. Pagitz et al. and, later, Gramüller et al. developed a bionically inspired approach based on the leaf movement of the Venus flytrap (Figure 1). The Venus flytrap is able to rapidly change the internal pressure within discrete areas of the trap leaves. This allows effective trapping and safe containment of insects and spiders. This approach was further developed so that, on the basis of an algorithm, the cross section of such pressure-actuated cellular structures (PACS) can be optimized with respect to a desired structural deformation by adjusting the length of the cell walls [10].

The result is the cross-sectional geometry of an optimized PACS, whose cells have a complex shape analogous to the cells of real plants and must exhibit extreme differences within the wall thickness for the necessary compliance (up to a factor of 12). For the production of the PACS, processes and materials are required with which the complex geometry specifications of the PACS cross section can be. As the first prototypes [9] have shown, the use of glass-fiber reinforced plastics (GFRP) is very promising here. However, the pre-impregnated fibers (prepregs) used for this must be manually placed in the form of the individual PACS cells. Automated deposition of the used prepregs within the cellular, non-planar geometry of the PACS is not possible. Manual positioning and draping of the prepregs prevent reproducible, precise adjustment of the wall thickness, which has a negative effect on the compliance characteristic. Furthermore, between adjacent cells is only a polymer-matrix-based connection. A continuous fiber-based connection of the cells to each other using prepregs is not feasible. As a result, PACS of this type have only limited resistance to fatigue strength. This type of production of complex PACS is, therefore, not scalable for high quantities or large components.

However, textile manufacturing technologies offer a high potential for the automation of preform operations for the production of complex fiber-reinforced polymer (FRP) structures with high reproducibility [12,13]. In comparison to different technologies such as knitting [14] and braiding [15], weaving is one of the most commonly used processes for the fabrication of textile-reinforced composite structures [16,17]. By integrating fiber reinforcement in a three-dimensional manner, which means in both in-plane and thickness directions [18], woven fabrics can be used as reinforcing structures in composites, which have a superior delamination resistance [19]. In addition, various approaches exist to provide fabrics with a cellular spacer structure in order to achieve a significant increase in stiffness and load-bearing capacity with a small increase in structural mass [20]. However, what these approaches have in common is that the compliance of the structures was not the driving factor in their development and, as structures with inherently too high stiffness, they are not suitable for implementing the basic principle of PACS. However, this does not mean that there are no actuated textile-reinforced composites [21,22]. There are so-called shape memory alloy fiber-reinforced polymers (SMA-FRPs), which can be activated via a textile-integrated SMA component (e.g., based on nickel and titanium) according to their compliance, which is also determined by their textile reinforcement structure. The mentioned SMA-FRP show great potential for the development of adaptive structures but have not yet been considered for transfer to the PACS principle in the context of the applications researched thus far.

**Figure 2:**
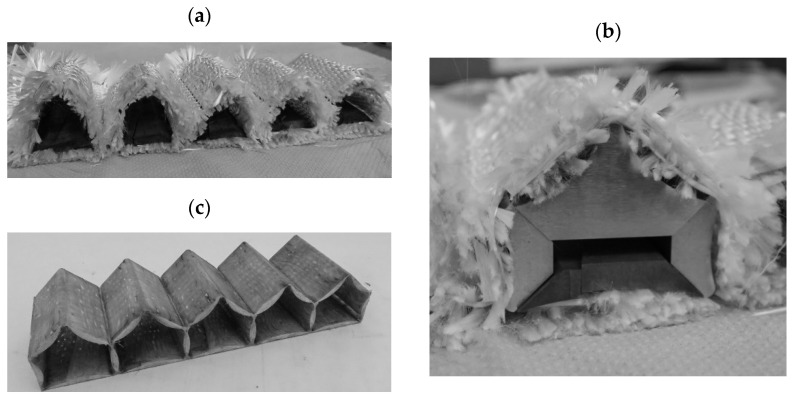
Textile reinforced single-row PACS: (**a**) woven fabric preform; (**b**) prepared preform with mold inlays before consolidation; (**c**) PACS composite.

A new approach for automated manufacturing using weaving technology was derived by Sennewald et al. [23]. This is based on the process combination of the technological approaches of terry weaving and spacer weaving [24]. For the woven textile PACS preform, an algorithm was developed to derive the simultaneously manufactured fabric layers under consideration of weaving technology restrictions. The high flexibility of the weaving technology enabled a continuous fiber-based connection of all PACS cells, as well as the representation of the required wall thickness gradient by process-integrated incorporation of solid pre-consolidated GFRP inlays. Using a subsequent process step to consolidate the woven preform in compression molding, new fabric-reinforced PACS could be developed but only in a single-row configuration [25,26] (see Figure 2).

This article represents the fundamental work and experiments to extend these findings regarding new double-row PACS, which enable a deflection not only in one direction but antagonistically adjustable deformation states. These are of great importance, for example, for the application in morphing wing concepts in order to specifically adapt the wing cross section for different operational conditions. This deformation behavior can be enabled analogously to the Venus flytrap by a double-row configuration of the PACS, whereby the cell rows can be separated and subjected to different internal pressures. Both for the necessary further development of the preform weave and for the technological implementation of the required geometry and wall thicknesses, the present results of single-row PACS can no longer be applied in a practicable way. The further development of the technological approach for double-row PACS requires extensive adaptation of weaving technology parameters (e.g., yarn arrangement, weave) and of the PACS cross section. These aspects are related in a complex way to the deformation behavior of the PACS and must therefore also be investigated in a complex manner.

**Figure 3:**
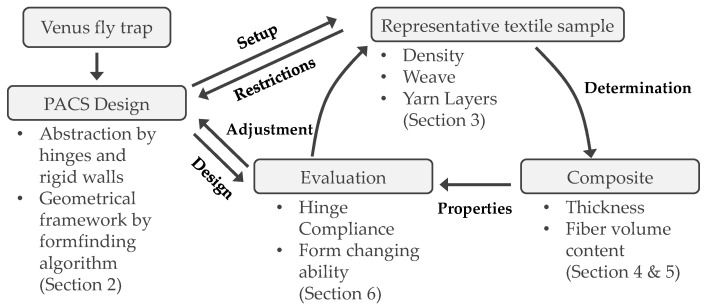
Process chain for the development and evaluation of textile, polymer composite, and design parameters for double-row PACS.

As a starting point for further development (Figure 3), the form-finding algorithm further developed by Meyer et al. is therefore used, on the basis of which a preliminary design approach for the cross section of double-row PACS can be derived. Based on the experimental results obtained in this study, this can be concretized up with information on the characteristics of the textile preform and the most important parameters of the achievable composite properties. A finite element (FE)-based simulation enables the iterative optimization of the textile and composite process parameters to be applied on the concretized cross-sectional design. For this purpose, the simulation results were compared with calculations for a design draft for equivalent PACS made of pure polyamides, such as a design that could be produced by selective laser sintering (SLS).

## 2. New Geometry Concept for Textile-Reinforced PACS in Antagonistic Configuration

The fundamental premise in the development of PACS is a cell cross section that remains constant along the spanwise direction. Therefore, all development steps can be performed using the 2D cross-sectional geometry. Compared to PACS in single-row configuration, the adequate determination of an optimized cell cross section is significantly more complex since the deformation behavior of the PACS is characterized by interactions between the cell rows. Furthermore, technological restrictions with regard to the weaving of the fabric preform, as well as the consolidation in compression molding, have to be taken into account. The creation of the PACS cross section is, therefore, described in detail in this section (Section 2.1), as well as its further development and modification (Section 2.2). Analogous to the implementation of single-row PACS, GFRP continues to be the material of choice due to its advantageous strength/stiffness ratio. In addition to the flexibly selectable geometrical design parameters (see Section 2.1), the mechanical parameters are the most important input variables for generating the cell cross sections. These were determined in advance of the design development using tensile specimens (DIN EN ISO 527, type 3) [27] from seven layers of orthotropic plain-woven fabric (Enka^®^ TecTape Hybrid Roving GF-PA 6733, warp and weft density 1.5/cm, further information in Section 4.1.1) with a total composite thickness of 2.15 mm in warp and weft direction. A universal testing machine from the manufacturer Instron (type 5584) with a 150 kN load cell was used as the testing device. The strain was measured using an extensometer. The evaluation of the tensile tests showed a Young’s modulus $E_{11}$ of 19,842 ± 458 MPa, a tensile strength of 375 ± 10 MPa, and an elongation at break of 2.65 ± 0.09% (confidence interval 95%). Since no significant difference could be found between the warp and weft testing direction, the material properties for both series of specimens were combined for statistical evaluation.

### 2.1. PACS Cross Section and Design

The basis for the development of the multi-row PACS with an antagonistic configuration of the cell rows and high deformation capacity is the form-finding algorithm described by Gramüller et al. [11]. The implementation of an improved cell design by Meyer et al. [28] also allows a better model prediction accuracy due to an optimized load-following alignment of the flexure hinges. Furthermore, a smooth and gap-free surface contour can now be realized, which is advantageous for aerodynamic applications. The underlying form-finding algorithm adapts the cell walls of a double-row structure of arbitrary polygonal cells in such a way that the structure can move between two predefined target shapes depending on its internal pressure. Based on geometric and performance-driven constraints, optimized coordinates for flexure hinges and cell walls were obtained, which could be used to derive the cross section of the PACS. The algorithm can be divided into two steps.

**Figure 4:**
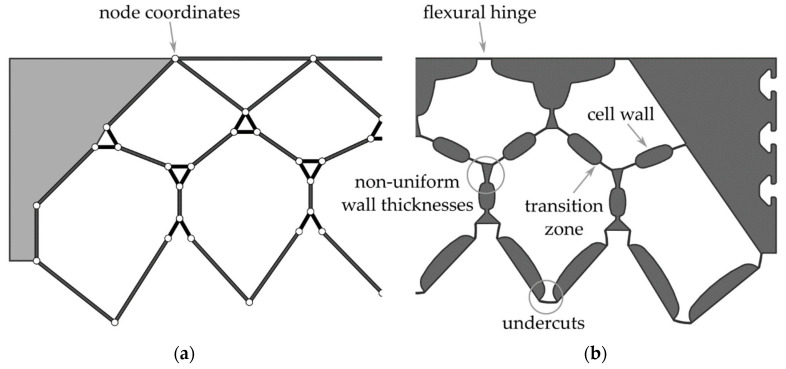
PACS design: (**a**) results from reduced-order truss model; (**b**) cross-sectional design optimized for additive manufacturing.

In a first step, the cell wall lengths are optimized on a reduced-order truss model with rigid cell walls and discrete point joints. Figure 4a shows the truss geometry obtained from the form-finding process for a double-row structure with four and five cells in the top and bottom cell rows, respectively. The following parameters were used as input for the form-finding process. These were derived from the size of the intended test structure:

- Cell width of 52 mm;
- Cell wall thickness of 6 mm;
- Flexure hinge thickness of 0.35 mm;
- Flexure hinge length of 5 mm;
- Flexure hinge eccentricity of 5 mm.

An internal pressure of 0.5 MPa and a deflection per cell of 5° were defined as performance-driven parameters. The input parameters were determined from boundary conditions of the weaving and consolidation process, as well as from previous experience in the textile–technical implementation of PACS [26].

In the second design step, the determined node coordinates are transformed into a cross section with solid flexure hinges, transition regions, and cell walls of specific thickness. Figure 4b shows the 2D cross section derived using the approach described in [11], which is optimized for manufacturing using additive manufacturing processes. However, a direct transfer of this cross-sectional geometry to FRP manufactured by the weaving process is not possible due to undercuts and non-uniform wall thicknesses. Therefore, a new approach to derive the cross-sectional geometry from the nodal coordinates has to be developed.

### 2.2. Cross-Sectional Adaption to FRP Process Restrictions

As a result of the algorithm, wall thicknesses and structural features of the preliminary optimized PACS cross section were distributed irregularly. In consequence, as a basis for the later weave development and consolidation tool design, the coordinates were aligned using a grid and a unified cell geometry. The efficiency of the weaving development for the woven fabric preform could thus be significantly increased, and representative weave areas could be identified for the necessary fundamental experimental work of this article. At the same time, a uniform structure of the PACS allowed a reduced effort in mold development so that the mold of the consolidation tool could later be created by assembling several identical parts and manufactured much more efficiently. Furthermore, areas of the PACS could be added that allowed for fiber-based coupling of cell rows, and potential undercuts could be eliminated. As a result, the post-processed coordinate set was used to develop a parameterized model for the PACS that includes the position of the flexure hinges with high accuracy and enables the adjustment of the wall thicknesses. Figure 4 shows a schematic depiction of the coordinate data set of the calculations and, based on it, the developed geometry of the PACS cross section.

**Figure 5:**
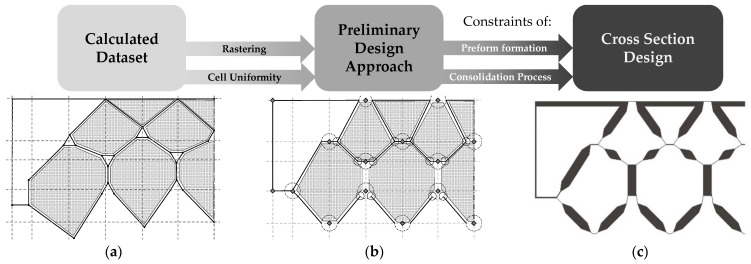
Steps of the geometry derivation for double row PACS (half-cross-sectional images of the symmetrical structure displayed): (**a**) calculated raw dataset and rastering; (**b**) processed dataset with aligned cell geometry; (**c**) derived cross section.

To generate the cross section, all relevant areas and structural features could be adjusted by the internal parameters of the model. This is of essential importance for the achievable thickness of the flexure hinges, the geometric expression of the cell areas, and the additionally required contact area between the cell rows. The additional contact areas can later be used to implement an interlaced fiber structure within the PACS composite regarding the fabric structure of the woven preform. Thus, delamination-induced fatigue failure of the PACS in this area can be avoided. The following experimental work thus serves to determine the parameters for a material- and process-suitable design of the flexure hinges, the cell walls, and the required contact areas (see Figure 5). Furthermore, for future research, it is planned to subsequently fill the V-shaped cavities above the contact areas with inlays, since these are not to absorb any deformation. The concretized design will be evaluated in an FE simulation with regard to the deformation capacity, and the cross-sectional design will be iteratively adapted.

## 3. Mechanical Properties of the Composite and the Weave Structure

The description of composites includes information about the reinforcing fiber and matrix components used and the arrangement in relation to the FRP component. In addition to these parameters, the fiber volume content $\rho_{F}$ determines the component properties. In terms of PACS, stiffness (Young’s modulus $E_{11}$) and tensile strength ($R_{11}$) in the fiber direction are crucial for targeted structural design. In advance of composite fabrication and without test data, these can be estimated using the mixing rules for the intended fiber–matrix combination [29]. In the first instance, these relationships were used for the calculation of preliminary geometry datasets in preparation for the development of the textile and PACS design.

(1) $$\rho_{C}=\rho_{F}\varphi_{C}+\rho_{M}\left(1-\varphi_{F}\right)$$

(2) $$E_{11}=E_{F}\varphi_{F}+E_{M}\left(1-\varphi_{F}\right)$$

(3) $$R_{11}=R_{F}\varphi_{F}+R_{M}\left(1-\varphi_{F}\right)$$

The deformation behavior of the PACS is determined by both Young’s modulus and material thickness t of the flexure hinge areas of the PACS. Material thickness is directly dependent on the characteristics of the fabric preform. Assuming mass equivalence of the fabric and composite, the material thickness $t_{C}$ in hinge areas can be determined as follows with knowledge of the areal density of the fabric preform $AD_{W}$:

(4) $$t_{C}=\frac{AD_{C}}{\rho_{C}}=\frac{AD_{W}}{\rho_{C}}=\frac{AD_{W}}{\rho_{F}\varphi_{F}+\rho_{M}\left(1-\varphi_{F}\right)}$$

**Figure 6:**
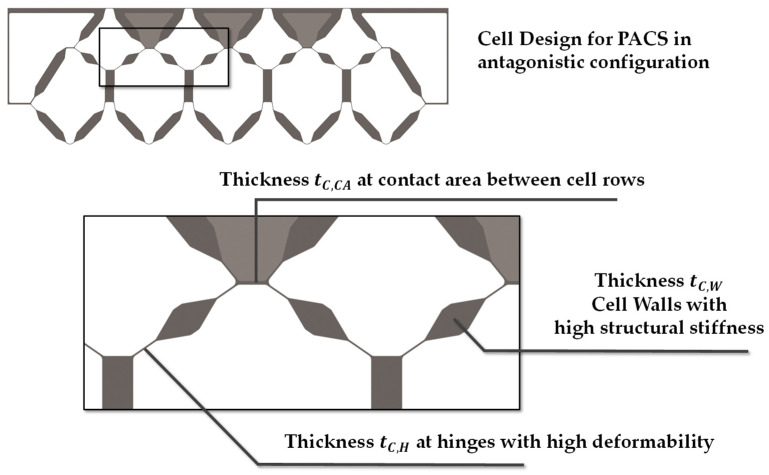
Cell design, geometrical characteristics, and thickness parameters.

The areal density of the fabric preform corresponds to the sum of the basis weights of the fabric layers, which can be determined in a sufficient approximation on the basis of the linear thread densities $t_{T,F}$ in warp and weft direction, as well as the average distance (see Figure 6) between the threads in warp and weft.

(5) $$AD_{W}=\overset{l_{W}}{\underset{l\;=\;1}{\sum }}\frac{t_{T,Warp,l}t_{T,Weft,l}}{a_{Weft,l}a_{Warp,l}}$$

With knowledge of the fabric weave and the average thread spacing within the preform layers, the area density can be calculated. The approach is based on the assumption that warp and weft yarns within the fabric have a quasi-circular cross section. In this way, it is possible to compare yarn positions available with actually occupied positions. Based on this assumption, it is then also possible to compare woven fabrics made of different materials and weaves [30,31]. Composites, on the other hand, are mostly composed of yarn materials with flat ribbon-like cross sections, and various modeling approaches exist to describe the yarn cross section (circular [32], racetrack [33], lenticular [34]). These allow accurate modeling and calculation of fabric properties in diverse applications (e.g., drape behavior, permeability) but are not practical for the intended evaluation of fabric properties for fabrication of the textile-generated and -reinforced PACS. Based on the material data of the reinforcement yarns, a substitute cross section was formed from several circular cross sections $d_{Warp,l,sub}$/$d_{Weft,l,sub}$ for the calculation of the fabric density $d_{W}$ and the fabric and material parameters (Figure 6).

Based on the definition of the equivalent cross sections, the relative fabric density $d_{W}$ is obtained as follows:

(6) $$d_{W}=\frac{p_{W}{\left(d_{Warp,l,sub}+d_{Weft,l,sub}\right)}^{2}}{a_{Weft,l,sub}a_{Warp,l,sub}}$$

Physically, the parameters fabric density and areal density/composite thickness are closely related. However, the direct analytical coupling is not readily possible. The reason for this is that the formulas mentioned do not contain any information about the porosity of the fabric preform. This varies considerably depending on the material and processing. Therefore, one objective of this work was the experimental determination of the compaction of the fabric preform during consolidation as a measure for the preform porosity. The compaction of the fabric preform during consolidation occurred in two stages (see Section 4). The first stage began with the closing of the mold. First, a hybrid yarn was used, which had a porosity of approx. 50–70%, and was compacted to the densest packing. A residual porosity remained within the preform, which was filled during the second phase. Under the influence of pressure and temperature, the polyamide component in the hybrid yarn melts and impregnates the glass filaments as the matrix. At the same time, further densification of the glass filaments occurs. The way in which stress within the preform is reduced by the melting of the polyamide of the hybrid yarn and the reinforcing fibers can thus assume the required position in the PACS in the composite according to the geometry of the mold. However, there are limits to this compaction due to the characteristic interlacing of the warp and weft yarns, since only yarn portions running parallel can be optimally compacted. Another compaction effect is represented by nesting, in which the proportion of crossing points that are shifted relative to one another and arranged one above the other enables additional compaction [35,36]. The determination and description of the compaction characteristics is, not at least, a decisive basis for the successful implementation of a tool design for the consolidation of the PACS.

## 4. Materials and Methods

### 4.1. Materials

#### 4.1.1. Basic Fabric Setup

**Figure 7:**
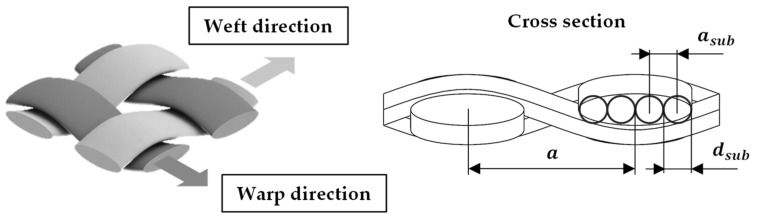
Yarn arrangement in woven fabric preforms (left) and cross-sectional view with substitute diameters.

In consequence, a certain main objective of this study was to determine the achievable wall thickness in the composite component as a function of the number of yarn layers and the fabric density. An *Enka^®^ TecTape Hybrid Roving GF-PA 6733* (PHP Fibers, Obernburg am Main, Germany) in warp and weft direction was used for fabric production. The roving has a linear density of 1855 tex with a glass fiber content of 67% by mass (corresponds to a volume content of approx. 46% in the composite). The sample was produced on a rapier loom *P1* from Lindauer DORNIER, Lindau, Germany. In particular, for the production of fabrics with several warp and weft layers (LTL1-3, Figure 7), this machine was equipped with a special thread take-up. Here, the warp threads run in successively staggered double flat steel heddles (e.g., *TWINtec* by DERIX, Grefrath, Germany), which is very advantageous when processing coarse warp material in high warp thread densities [37].

On the one hand, single-layer samples of the respective fabric configuration were investigated and, on the other hand, multilayer stacked samples. In this way, the resulting composite wall thickness in each case is determined using 2 up to 10 fabric layers stacked on top of each other. The structure of the individual layers investigated and an overview of the fabric configurations are shown in Figure 5 and Table 1.

#### 4.1.2. Test Series and Processing

According to the objective of analyzing the influence of weft density and the number of yarn plies, two different sample series were used. On the one hand, single-ply plain weaves with different weave densities, and on the other hand, fabric stacks from different weaves were investigated (Table 2).

**Figure 8:**
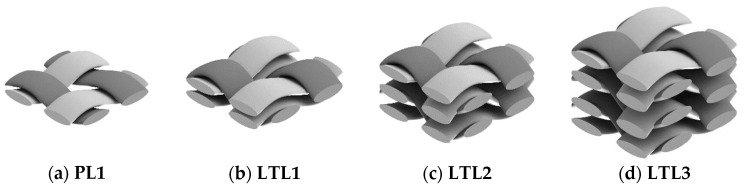
Overview of the basic fabric weave and their structure: (**a**) plain-woven fabric; (**b**–**d**) layer-to-layer weaves.

For the systematic investigation of the composite wall thickness as a function of the number of layers (samples No. 1, 6–16), various combinations are possible with the given weaves (Figure 8). These can be formed on the basis of the partitioning (summation decomposition) [38] of the required number of layers. To limit the testing effort, technologically equivalent variants were selected with respect to the number of plies so that the yarn plies 2 to 10 were represented at least once and, for control purposes, the number of layers 5 and 7 were represented twice (Table 3).

**Figure 9:**
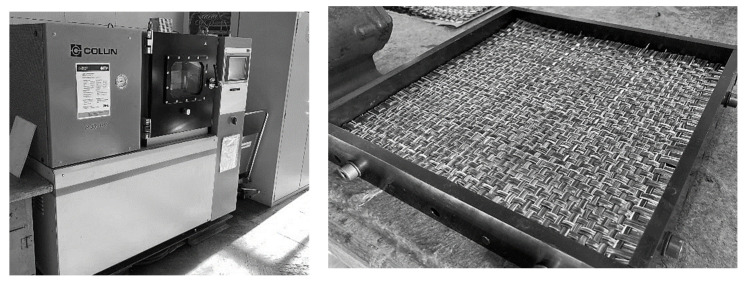
Sample consolidation: laboratory platen-press COLLIN 300 PV and plate tool.

**Figure 10:**
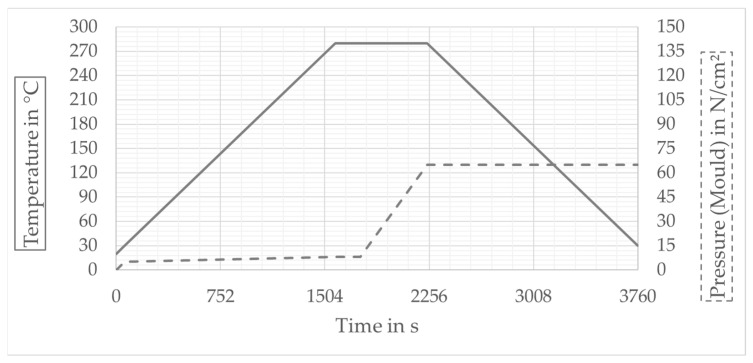
Temperature–pressure–time diagram of the sample consolidation.

The processing of all composite specimens was carried out by compression molding on a *COLLIN P300 PV* laboratory heating press (COLLIN Lab & Pilot Solutions, Maitenbeth, Germany) in a platen tool (Figure 9). The temperature–pressure–time cycle during specimen consolidation was matched to the processing of the PA6 matrix, ensuring full impregnation of the glass fibers and a minimum pore content (Figure 10, maximum temperature 280 °C, maximum pressure 65 N/cm^2^, heating and cooling rate 10 K/min). The consolidation process was carried out in a residual ambient pressure of 0.01 MPa.

### 4.2. Methods

All measurements on textile and composite samples were carried out under standard climate conditions (DIN EN ISO 139: 20 °C, 65% rel. humidity) [39] and after a minimum of 48 h sample conditioning, in order to establish an equilibrium with regard to the water absorption of polyamide from the environment.

**Figure 11:**
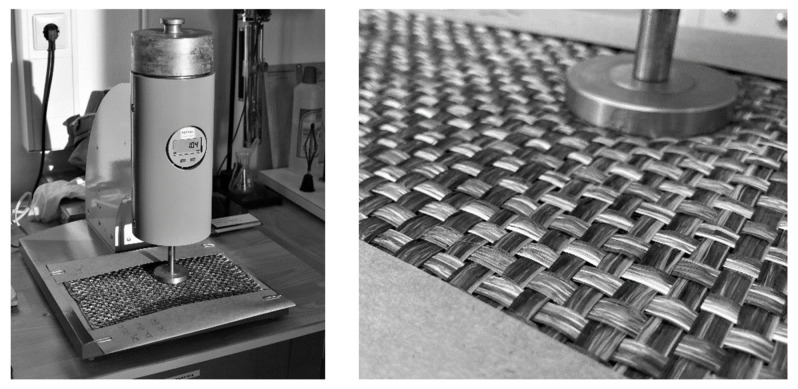
Thickness measurement device rainbow (KARL SCHRÖDER/SYLVAC).

The fabric thickness was measured with a *Rainbow* digital textile thickness tester from KARL SCHRÖDER (Weinheim, Germany) in combination with a digital measuring device (Sylvac S229, SYLVAC, Yverdon, Schwitzerland, Figure 11) according to DIN EN ISO 139 [40] (measuring plunger pressure area 25 cm^2^, test pressure 2 kPa). The measurements were made 30 s after specimen gripping. Eight measured values were distributed over the specimen areas, and the average material thickness was determined from them. Paper support (measured: thickness 0.3 mm; area density 0.231 kg/m^2^) was used to transfer the specimens between cutting, measurement, and consolidation, and its values were subtracted from the measured values in the evaluation. The consolidated samples were also tested according to the same methodology. Furthermore, the fiber volume content was determined on random samples according to DIN EN ISO 1172 [41]. The areal density was determined gravimetrically on all samples.

## 5. Results

**Figure 12:**
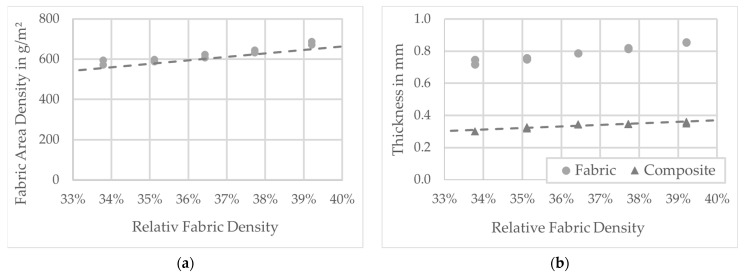
Results of the fabric testing for various weft yarn densities: (**a**) fabric area density and calculation result as trendline; (**b**) fabric and composite thickness with calculation result as the trendline.

**Figure 13:**
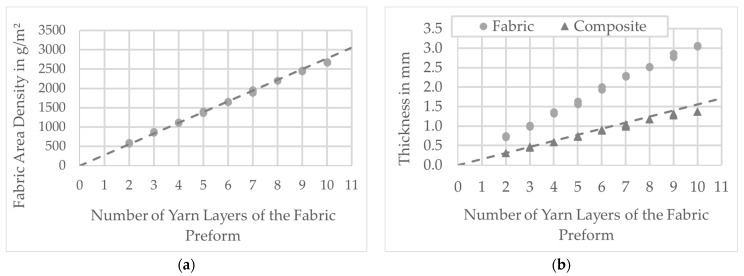
Results of the fabric testing for different number of yarn layers of the preform: (**a**) fabric area density and calculation result as trendline; (**b**) fabric and composite thickness and calculation result as the trendline.

The diagrams (Figure 12 and Figure 13) show the results of the tests. The diagrams in Figure 11 show the results of the investigation of fabric preforms with the same ply structure and fabric weave. To analyze the influence of relative fabric density on fabric and composite thickness, the weft density was increased successively (see Table 1 and Table 2). In addition to the measured data, the results of the calculations (according to Equations (4) and (5)) are also entered. The comparison shows that measurement and calculation agree well. Figure 13 shows the results of varying the layer structure of the preforms by successively increasing the number of yarn layers. The resulting increase in composite wall thickness is clearly visible. On the one hand, the comparison of the measured values with the results of the calculations shows that, especially for preform structures with a low number of plies, measurement and calculation agree well with each other. On the other, with increasing preform thickness, an increasing deviation between measured and calculated values can be observed.

As already shown in Table 3, the greater the number of layers, the greater the possibilities of forming them from the different fabric layers within the preform. However, the measured values do not show any effects of the arrangement of the fabric layers on the preform and composite thickness. Thus, the basic assumption can be confirmed that, with similar preform surface mass, it is above all the number of superimposed yarn layers that determines the composite thickness. Thus, preform thickness and composite thickness can be defined with a high degree of certainty as decisive factors for the detailed PACS consolidation tool design. Figure 13 shows the average fiber volume content of all samples.

## 6. Discussion

Based on the experimental results, the design for integrally fabric-reinforced PACS can now be concretized and the deformation capacity can be adequately evaluated. The determined correlations between the fabric preform and composite design are presented and discussed together with the results for the simulation of the deformation capacity.

### 6.1. Weave Density and Preform Layup

The deformation behavior and load capacity of the PACS are highly dependent on the wall thickness within the flexure hinge areas. The wall thickness in the hinges determines the stress that can be applied normally in the direction of the hinge by applying pressure to the cells. The capacity to change, on the other hand, is determined by the bending stiffness of the hinges (see [16]). In order to match the fundamental design framework for the flexure hinges with a length of about 5 mm within the fabric preform, it is necessary to increase the weft density and thus the fabric density from approx. 34% to approx. 39%. For this purpose, concrete values for the expected wall thicknesses within the hinges could be obtained on the basis of the tests. Thus, an increase in the weft density from 1.5/cm to 2/cm results in an equally approx. 18% higher wall thickness in the hinge from 0.30 mm to approx. 0.35 mm. Based on the measurement results and the calculations, a directly proportional relationship between fabric density and composite wall thickness for the thin-walled hinges can be demonstrated with these investigations (assumption: mass equivalence of preform and composite).

**Figure 14:**
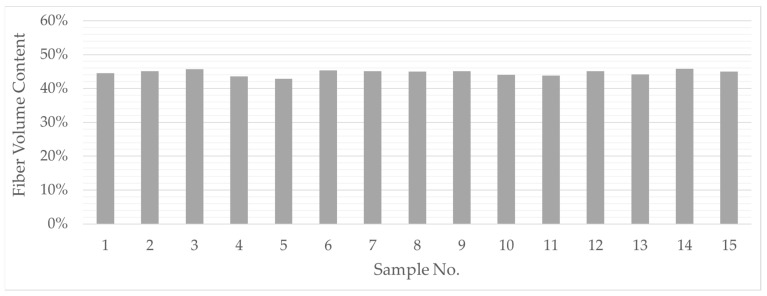
Overview of the measured values for the fiber volume content.

Another factor influencing the thickness of the composite is the sequencing of the layers within the fabric preform. According to Table 3, the preform structure can consist of individual layers with different bond-determined numbers of filaments. A fundamental characteristic of the compaction and consolidation behavior of the fabric preforms is that crossed filament layers “lock” each other, i.e., glass filaments of a yarn system cannot be displaced into adjacent, parallel yarns during consolidation. This relationship can be observed on the basis of the experimental analysis of the fiber volume content. The share of reinforcing fibers is at the very same level of approx. 45% for all samples, which corresponds to the fiber volume content of the hybrid polyamide/glass raw material. The test results showed that this effect is also effective when stacking different individual layers up to a certain preform thickness. Irrespective of the weave, preforms with cumulatively the same number of filament layers (e.g., test series no. 11: 1 × 2 + 1 × 5 vs. test series no. 12: 1 × 7) exhibit similar compacting behavior (see Figure 14).

Above a yarn layer count of 9, an increasing deviation of the composite thickness between the calculated values and the measured values can be observed (e.g., test series 15 with 14% difference). This is due to increasing nesting effects between the preform layers. The decisive factor for the occurrence of the overall composite thickness is the concentration of statistically distributed areas with parallel oriented filament layers within the individual preform layers. During consolidation, these areas are locally more densified, so that the expected proportional increase in the composite wall thickness is then no longer given. This was taken into account when deriving the mold geometry of the cell wall area.

### 6.2. Preform and Consolidated PACS

**Figure 15:**
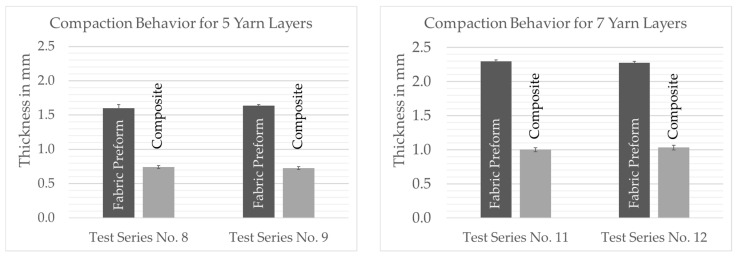
Comparison between different layup variants and preform compaction behavior during consolidation.

The measurement results allow the preform design to be specified and the resulting PACS geometry to be estimated with a high degree of accuracy and certainty. Compared to other thermoplastic reinforcement materials, the used TecTape shows a significantly reduced volume shrinkage of less than 50% in the consolidation (See Figure 15). For comparable thermoplastic materials, this is 70% and more [42]. This finding significantly simplifies the development of a PACS consolidation tool, since the measures required to compensate for volume shrinkage can be much lower. Furthermore, the determination of values and basic assumptions for the estimation of the deformation of the PACS for the simulation are possible with a high degree of certainty.

**Figure 16:**
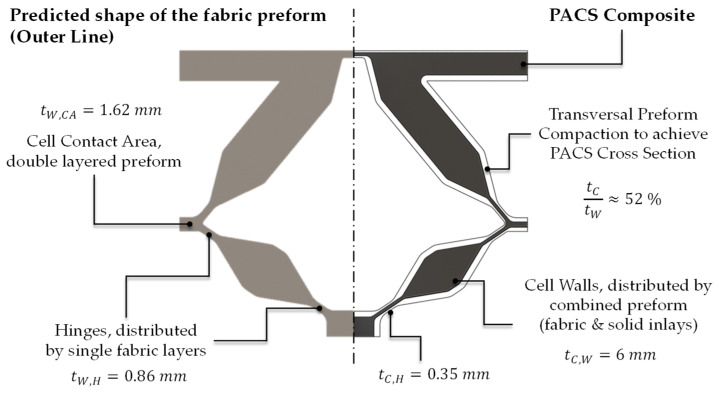
Cross section of the fabric preform and the consolidated PACS.

The realization of the necessary maximum length of the hinges of 5 mm requires a weave density of the preform of 39%. This results in a thickness of the woven preform of approx. 0.86 mm and the consolidated PACS of 0.35 mm in the hinge areas. This value is significantly lower than the one reported in [29] of approx. 0.5 mm. This improvement can be attributed to the modified production setup, which had to be adapted to the production of the double-row PACS. On the other hand, interlacement influences can consequently be regarded as quasi-homogeneously distributed over the cross section of the flexure hinges. The contact area between the hinges (see Figure 6 and Figure 16) must be double layered for a stable coupling between the cell rows. This is achieved in the production of the PACS preforms by weaving the upper and middle warp layers interlocked together. This avoids shear and cross-tension-induced delamination as a result of the large structural distortion during the actuation of the PACS. The thickness of the PACS preform is 1.62 mm in the contact areas and approx. 0.74 mm after consolidation.

The overall stiffness of the PACS is adjusted via the thickness of the cell walls. For a secure closure [26], the cell walls should simultaneously be used for screwing on appropriate closure caps. These can be realized by weaving-integrated inserts or screws directly inserted into the composite. The implementation will be concretized in the context of the pending tool development for the consolidation of the new PACS. The target value for the geometry development of the PACS is therefore a composite wall thickness of 6 mm. With linear regression of the measured values, the fabric preform would have to consist of 39 filament layers and have a thickness of 11.7 mm. Since the influence of nonlinear nesting effects increases significantly with the number of yarn layers, this value will be even greater in reality. The purely weaving implementation of such thicknesses is not reasonable. Nevertheless, in order to represent such wall thicknesses, different strategies are available, including monolithic composite using composite inserts [25,26], sandwich, or hollow chamber structure. The selection was made considering the results of the numerical evaluation of the deformation behavior.

### 6.3. Evaluation of the PACS Deformation Behavior

**Figure 17:**
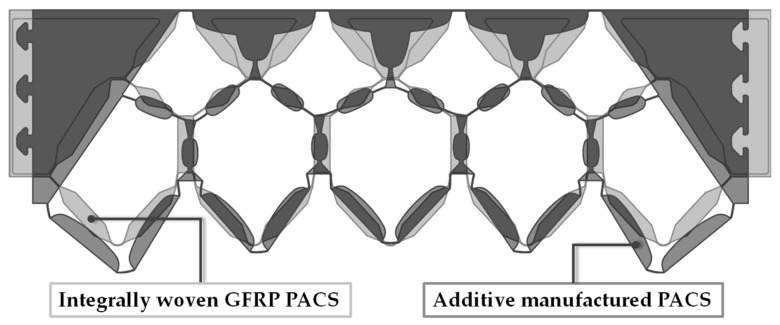
Comparison of two PACS cross-sectional designs: “CS-AM” optimized for additive manufacturing; “CS-FRP” adapted to restrictions from integral woven GFRP manufacturing.

A finite element analysis (FEA) was used to evaluate the deformation capacity of the PACS. Two cross-sectional designs were investigated: (i) “CS-AM”, with the cross section optimized for additive manufacturing (AM) according to [11] (Figure 4b); (ii) “CS-FRP”, with the design adapted to integral woven GFRP manufacturing (Figure 5c). An overlay of both designs is shown in Figure 17.

For the FEA, a 2D model was used in ANSYS assuming a plane strain state. The meshing was performed with quadratic plane-183 elements. The mesh density was controlled by the number of elements across the gap. In a mesh refinement study, convergence was obtained for six elements in the thickness direction of the flexure hinges, resulting in a total of 38,053 elements for “CS-AM” and 29,975 elements for “CS-FRP”. The internal pressure was applied via surf153 elements as a surface effect.

**Figure 18:**
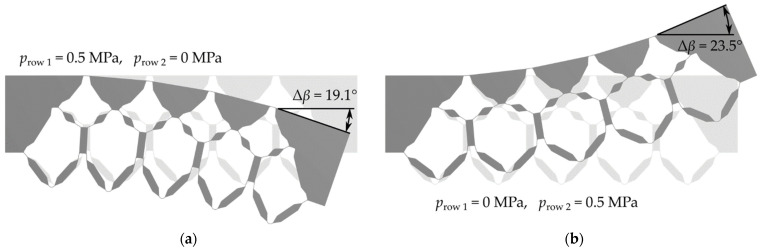
Deflection of “CS-FRP” calculated with FEA for both targeting states: (**a**) pressurization of the upper cell row; (**b**) pressurization of the lower cell row.

Figure 18 shows the cross-sectional design of “CS-FRP” in the deformed state for the upper and lower deflection when only one cell row was pressurized with the design pressure of 0.5 MPa in each case. For the calculations, the upper triangles and the two outer connection elements were considered as solid materials, since they do not contribute to the compliance and are later filled with inlays. The left edge of the model was fixed. The angular displacement Δβ of the right outer edge was considered as a measure of the change in shape. By selectively adjusting the pressure difference between the two cell rows, any deflections between the two extreme states could be achieved.

**Figure 19:**
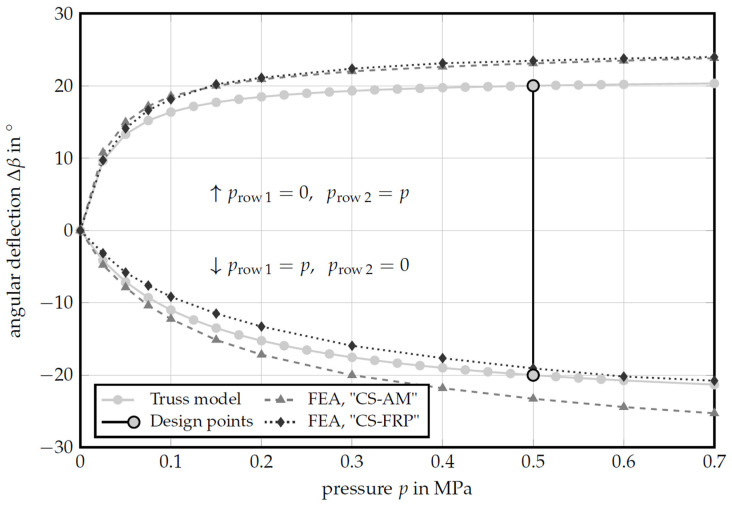
Angular deflection Δβ as a function of the cell pressure. Comparison of the integrally woven “CS-FRP” and the “CS-AM” optimized for additive manufacturing. Pressurization was applied to only one cell row at a time, while the pressure in the other was set to zero.

Figure 19 shows the mechanical behavior of the cellular structure as a function of the internal pressure when only one cell row was pressurized. The results from the reduced-order truss model were compared with the FEA results for the two cross-sectional designs “CS-AM” and “CS-FRP”. Differences between the reduced-order truss model and the FEA on the design “CS-AM” have already been explained in [26] and arise primarily from the simplification in the reduced-order model. The hinge transition areas were not taken into account and the deformation of the cell walls was neglected, both resulting in lower deflections, compared to the FEA.

A comparison of the two cross-sectional designs “CS-AM” and “CS-FRP” shows that pressurizing the lower cell row results in an almost identical deformation curve for both designs. Various restrictions had to be taken into account when converting the cross section into a weave- and tool-compatible cross-sectional design (Section 2.2). As a result of the simulation, it can be shown that a simplification of the cross-sectional geometry does not have to result in any drawbacks with regard to the deformation capacity restrictions of the compliant mechanism of the PACS.

When the upper cell row is pressurized, the “CS-FRP” design appears stiffer than “CS-AM”. This deviation needs further investigation. Currently, it is assumed that it results from the unification of the cells and the nodal coordinates. This leads to a reduced eccentricity of the undeformed upper cell in this row and to a reduction of the deformation capacity. In general, the upper cell row reacts more sensitively to changes in the loading condition, since the lever arm is lower relative to the bending neutral axis.

## 7. Conclusions

The presented work established the essential basis for the development of integrally textile-reinforced double-row PACS. In the beginning, it was possible to draw on extensive empirical data, which enabled the successful previous development of textile-reinforced single-row PACS. In the course of the consistent extension of the PACS approach to double-row structures, it became apparent that the technological and theoretical bases used thus far were not sufficient to capture and produce the significantly more complex geometry of double-row PACS. As a result, a new PACS design was derived on the basis of existing experience in the development of single-row PACS and an extended form-finding algorithm and was further developed for implementation as a textile-reinforced FRP structure. An essential criterion in deriving the highest possible deformability of the PACS is the compliance of the flexure hinges, the material-dependent stiffness, elongation limit, and, last but not least, the wall thickness as a geometry-based coefficient. On the basis of the new experimental data, it could be shown that the latter in particular can be significantly improved with the use of a new setup in fabric production, whereby the wall thickness can be reduced from the previous 0.5 mm to the current 0.35 mm. This corresponds to a reduction of 30% and, in terms of hinge stiffness, approx. 65%, which means that the potential deformation capacity of the PACS can be significantly increased in the future. The experimental investigations identified a strict relationship between hybrid yarn material, fabric weave, and the resulting composite attributes. Consequently, the knowledge about these findings enables the effective further development of fabric structure for integrally woven double-row PACS. The textile design is strongly influenced by the properties of the subsequent composite structure, in particular the wall thickness within the flexure hinges. These influences and essential correlations are now more describable. In connection with the further developed unified and adapted design, the tool design for the consolidation of the PACS preform can be derived. Numerical simulations prove that these necessary adaptations do not cause significant disadvantages for the later form-changing capability of the PACS and that a high degree of functionality can be achieved by the integrally woven fabric-reinforced PACS. These results are a significant milestone in the development of textile-reinforced PACS. Further studies will therefore be carried out to validate the mechanical model assumptions for flexure properties of flexure hinges based on plain-woven fabric reinforcement. This will be carried out experimentally using a test method close to the component and published in a near future. Based on the collected data and simulative evaluation of the current geometry design of the PACS, it is now possible to develop a concrete geometry and component-compatible design of the required fabric weaves. Last but not least, the finalized PACS geometry will be used to design a further developed tool. This concerns in particular the design of the cell rows and the necessary displacement of the mold inserts for axis-appropriate compression and consolidation of the preform.

## Figures and Tables

**Table 1:** Overview of examined woven fabric preform configurations.

Weave Setup	Number of Yarn Layers		Number of Warp Yarns per cm		Number of Weft Yarns per cm	
	Warp	Weft	Per Layer	∑	Per Layer	∑
PL1	1	1	1.50	1.50	1.50	1.50
PL1a	1	1	1.50	1.50	1.62	1.62
PL1b	1	1	1.50	1.50	1.74	1.74
PL1c	1	1	1.50	1.50	1.86	1.86
PL1d	1	1	1.50	1.50	2.00	2.00
LTL1	1	2	1.50	1.50	1.50	3.00
LTL2	2	3	1.50	3.00	1.50	4.50
LTL3	3	4	1.50	4.50	1.50	6.00

**Table 2:** Test series numbering and assignment.

Examined Parameter	Series Identification No.	Weave Setup
Relative Weave Density	1	PL1
	2	PL1a
	3	PL1b
	4	PL1c
	5	PL1d
Number of Yarn Layers	1 *; 6–16	various (see Table 3)

* Series no. 1 has been used for both examination objectives.

**Table 3:** Selection of possible partitioning options in the structure of the fabric samples with 2 to 10 yarn layers. The examined variants are marked with x. The numbering is consecutive for the identification of the respective sample.

Preform Layer	Number of Yarn Layers of the Preform																					
	2	3	4	5	5	6	6	7	7	7	8	8	8	9	9	9	9	10	10	10	10	10
2/(a) **	1		2		1	3		2	1		4	1		3		2	1	5	2	1		
3/(b)		1			1		2	1				2	1	1	3				2	1		1
5/(c)				1					1				1			1				1	2	
7/(d)										1							1					1
Tested:	x	x	x	x	x	x			x	x	x			x						x		
**No.:**	**1**	**6**	**7**	**8**	**9**	**10**			**11**	**12**	**13**			**14**						**15**		

** according to Figure 1.

## Abbreviations

The following abbreviations are used in this manuscript:

AM	Additive manufactured
CS-AM	Cellular structure—additive manufactured
CS-FRP	Cellular structure—Fiber-reinforced plastic
FE	Finite elements/Finite elements analysis
GF	Glass Fiber
GFRP	Glass-fiber-reinforced plastic
LTL	Layer-to-layer woven fabric
PA	Polyamide
PACS	Pressure-actuated cellular structure
PL	Plain-woven fabric
SLS	Selective laser sintering
SMA	Shape memory alloy

## References

[1] Howell L.L., Magleby S.P., Olsen B.M. (2013). Handbook of Compliant Mechanisms.

[2] Dresig H., Vul’fson I.I. (1989). Kinematik zwangläufiger Mechanismen. Dynamik der Mechanismen.

[3] Shuib S., Ridzwan M., Kadarman A.H. (2007). Methodology of Compliant Mechanisms and its Current Developments in Applications: A Review. Am. J. Appl. Sci..

[4] Kota S., Flick P., Collier F.S. (2016). Flight Testing of FlexFloil^TM^ Adaptive Compliant Trailing Edge. 54th AIAA Aerospace Sciences Meeting.

[5] Elzey D.M., Sofla A.Y.N., Wadley H.N.G., Lagoudas D.C. (2003). A bio-inspired, high-authority actuator for shape morphing structures. Proceedings of the Smart Structures and Materials 2003: Active Materials: Behavior and Mechanics.

[6] Vasista S., Titze M., Schäfer M., Bertram O., Riemenschneider J., Monner H.P. (2020). Structural and Systems Modelling of a Fluid-driven Morphing Winglet Trailing Edge. Proceedings of the AIAA Scitech 2020 Forum.

[7] Pagitz M., Lamacchia E., Hol J.M. (2012). Pressure-actuated cellular structures. Bioinspir. Biomim..

[8] Luo Q., Tong L. (2013). Adaptive pressure-controlled cellular structures for shape morphing I: Design and analysis. Smart Mater. Struct..

[9] Gramüller B., Boblenz J., Hühne C. (2014). PACS—Realization of an adaptive concept using pressure actuated cellular structures. Smart Mater. Struct..

[10] Gramüller B., Köke H., Hühne C. (2015). Holistic design and implementation of pressure actuated cellular structures. Smart Mater. Struct..

[11] Sinapius M., Hühne C., Sadri H., Riemenschneider J. (2021). Active Shape Control. Adaptronics—Smart Structures and Materials.

[12] Cherif C. (2016). Textile Materials for Lightweight Constructions—Technologies, Methods, Materials, Properties.

[13] Chen X., Taylor L.W., Tsai L.-J. (2011). An overview on fabrication of three-dimensional woven textile preforms for composites. Text. Res. J..

[14] Chen F., Hu H., Liu Y. (2015). Development of weft-knitted spacer fabrics with negative stiffness effect in a special range of compression displacement. Text. Res. J..

[15] Rawal A., Saraswat H., Sibal A. (2015). Tensile response of braided structures: A review. Text. Res. J..

[16] Ansar M., Xinwei W., Chouwei Z. (2011). Modeling strategies of 3D woven composites: A review. Compos. Struct..

[17] Mountasir A., Hoffmann G., Cherif C., Kunadt A., Fischer W.-J. (2012). Mechanical characterization of hybrid yarn thermoplastic composites from multi-layer woven fabrics with function integration. J. Thermoplast. Compos. Mater..

[18] Boussu F., Cristian I., Nauman S. (2015). General definition of 3D warp interlock fabric architecture. Compos. Part B Eng..

[19] Tripathi L., Behera B.K. (2021). Review: 3D woven honeycomb composites. J. Mater. Sci..

[20] Mountasir A., Löser M., Hoffmann G., Cherif C., Großmann K. (2016). 3D Woven Near-Net-Shape Preforms for Composite Structures. Adv. Eng. Mater..

[21] Bollas D., Pappas P., Parthenios J., Galiotis C. (2007). Stress generation by shape memory alloy wires embedded in polymer composites. Acta Mater..

[22] Ashir M., Hindahl J., Nocke A., Cherif C. (2019). A statistical approach for the fabrication of adaptive pleated fiber reinforced plastics. Compos. Struct..

[23] Sennewald C., Vorhof M., Schegner P., Hoffmann G., Cherif C., Boblenz J., Sinapius M., Hühne C. (2018). Development of 3D woven cellular structures for adaptive composites based on thermoplastic hybrid yarns. IOP Conf. Ser. Mater. Sci. Eng..

[24] Mountasir A., Hoffmann G., Cherif C., Löser M., Großmann K. (2015). Competitive manufacturing of 3D thermoplastic composite panels based on multi-layered woven structures for lightweight engineering. Compos. Struct..

[25] Sennewald C., Vorhof M., Hoffmann G., Cherif C. (2018). Overview of Necessary Development Steps for the Realization of Woven Cellular Structures for Adaptive Composites. J. Fash. Technol. Text. Eng..

[26] Meyer P., Boblenz J., Sennewald C., Vorhof M., Hühne C., Cherif C., Sinapius M. (2019). Development and Testing of Woven FRP Flexure Hinges for Pressure-Actuated Cellular Structures with Regard to Morphing Wing Applications. Aerospace.

[27] Deutsches Institut für Normung e.V. (2003). DIN EN ISO 527: Kunststoffe—Bestimmung der Zugeigenschaften.

[28] Meyer P., Lück S., Spuhler T., Bode C., Hühne C., Friedrichs J., Sinapius M. (2021). Transient Dynamic System Behavior of Pressure Actuated Cellular Structures in a Morphing Wing. Aerospace.

[29] Schürmann H. (2007). Konstruieren Mit Faser-Kunststoff-Verbunden.

[30] Walz F., Luidbrandt J. (1947). Die Gewebedichte I. Textilpraxis.

[31] Walz F., Luidbrandt J. (1947). Die Gewebedichte II. Textilpraxis.

[32] Peirce F.T. (1937). The Geometry of Cloth Structure. J. Text. Inst. Trans..

[33] Kemp A. (1958). An Extension of Peirce's Cloth Geometry to the Treatment of Non-circular Threads. J. Text. Inst. Trans..

[34] Hearle J.W.S., Shanahan W.J. (2008). An Energy Method for Calculations in Fabric Mechanics, Part I: Principles of the Method. J. Text. Inst..

[35] Lomov S.V., Verpoest I., Peeters T., Roose D., Zako M. (2003). Nesting in textile laminates: Geometrical modelling of the laminate. Compos. Sci. Technol..

[36] Potluri P., Sagar T.V. (2008). Compaction modelling of textile preforms for composite structures. Compos. Struct..

[37] Vorhof M., Weise D., Sennewald C., Hoffmann G. (2020). New method for warp yarn arrangement and algorithm for pattern conversion for three-dimensional woven multilayered fabrics. J. Ind. Text..

[38] Steger A. (2007). Diskrete Strukturen.

[39] Deutsches Institut für Normung e.V. (2011). DIN EN ISO 139: Textilien—Normalklimate für die Probenvorbereitung und Prüfung.

[40] Deutsches Institut für Normung e.V. (1996). DIN EN ISO 5084: Bestimmung der Dicke von Textilien und Textilen Erzeugnissen.

[41] Deutsches Institut für Normung e.V. (1998). DIN EN ISO 1172: Prepregs, Formmassen und Laminate—Bestimmung des Textilglas- und Mineralfüllstoffgehalts.

[42] FOREL—3DProCar Bericht. https://plattform-forel.de/3dprocar-bericht/.

